# Atrial Rotor Dynamics Under Complex Fractional Order Diffusion

**DOI:** 10.3389/fphys.2018.00975

**Published:** 2018-07-24

**Authors:** Juan P. Ugarte, Catalina Tobón, António M. Lopes, J. A. Tenreiro Machado

**Affiliations:** ^1^Grupo de Investigación en Modelamiento y Simulación Computacional, Facultad de Ingenierías, Universidad de San Buenaventura, Medellín, Colombia; ^2^MATBIOM, Universidad de Medellín, Medellín, Colombia; ^3^UISPA-LAETA/INEGI, Faculty of Engineering, University of Porto, Porto, Portugal; ^4^Department of Electrical Engineering, Institute of Engineering, Polytechnic of Porto, Porto, Portugal

**Keywords:** rotor dynamics, atrial fibrillation, complex order diffusion, structural heterogeneity, electrical remodeling

## Abstract

The mechanisms of atrial fibrillation (AF) are a challenging research topic. The rotor hypothesis states that the AF is sustained by a reentrant wave that propagates around an unexcited core. Cardiac tissue heterogeneities, both structural and cellular, play an important role during fibrillatory dynamics, so that the ionic characteristics of the currents, their spatial distribution and their structural heterogeneity determine the meandering of the rotor. Several studies about rotor dynamics implement the standard diffusion equation. However, this mathematical scheme carries some limitations. It assumes the myocardium as a continuous medium, ignoring, therefore, its discrete and heterogeneous aspects. A computational model integrating both, electrical and structural properties could complement experimental and clinical results. A new mathematical model of the action potential propagation, based on complex fractional order derivatives is presented. The complex derivative order appears of considering the myocardium as discrete-scale invariant fractal. The main aim is to study the role of a myocardial, with fractal characteristics, on atrial fibrillatory dynamics. For this purpose, the degree of structural heterogeneity is described through derivatives of complex order γ = α + *jβ*. A set of variations for γ is tested. The real part α takes values ranging from 1.1 to 2 and the imaginary part β from 0 to 0.28. Under this scheme, the standard diffusion is recovered when α = 2 and β = 0. The effect of γ on the action potential propagation over an atrial strand is investigated. Rotors are generated in a 2D model of atrial tissue under electrical remodeling due to chronic AF. The results show that the degree of structural heterogeneity, given by γ, modulates the electrophysiological properties and the dynamics of rotor-type reentrant mechanisms. The spatial stability of the rotor and the area of its unexcited core are modulated. As the real part decreases and the imaginary part increases, simulating a higher structural heterogeneity, the vulnerable window to reentrant is increased, as the total meandering of the rotor tip. This *in silico* study suggests that structural heterogeneity, described by means of complex order derivatives, modulates the stability of rotors and that a wide range of rotor dynamics can be generated.

## 1. Introduction

Atrial fibrillation (AF) represents an important socio-economic burden for world health systems (Kirchhof et al., 2016). Research efforts are focused on determining the AF underlying mechanisms (Zaman and Peters, 2014). Catheter ablation has improved the outcomes of therapeutic interventions for patients in early stages of the arrhythmia (Haïssaguerre et al., 1998; Atienza et al., 2014). However, as the AF perdures in time, the ablation effectiveness decreases significantly (Kirchhof et al., 2016; Lim et al., 2017). During this chronic AF (CAF) scenario, the pathophysiological substrate sustains a more complex form of the arrhythmia. The rotor hypothesis establishes that an AF episode is sustained by a single or several spiral waves known as rotors, activating the surrounding tissue at high rates and generating complex patterns of propagation (Jalife et al., 2002). A rotor is a functional reentry that circumvolves an excitable but unexcited core (Guillem et al., 2016). Recent clinical studies report high rates of success when targeting rotors as ablation sites in CAF patients (Narayan et al., 2012, 2013; Miller et al., 2017). This investigation provides evidence in favor of the rotor hypothesis, but controversy persists since some researchers were not able to replicate the results (Buch et al., 2016; Steinberg et al., 2017). Therefore, a better understanding of the rotor dynamics and the effect of structural heterogeneity, could lead to deeper knowledge for determining critical ablation targets.

The electrical and structural remodeling that the atrial tissue undergoes during CAF, yield a complex interplay in sustaining the arrhythmia (Trayanova et al., 2014). It is recognized that abnormal structural changes play a larger role in perpetuating of CAF than the electrical remodeling alone (Anné et al., 2007). Electrophysiological models were used for understanding the start-up and the perpetuation of rotors, since this task is not easy to develop in experimental terms. The proposed computational descriptions of rotors propagating in a structurally remodeled atrial tissue, provided insight of how rotors evolve under such circumstances (Trayanova et al., 2014; Zhao et al., 2015; Hansen et al., 2017). Structural heterogeneities are modeled through non-conducting elements, reduced conductivity elements and boundary conditions. However, a precise knowledge of tissular conditions is needed in order to set the model parameters (Stinstra et al., 2010). Furthermore, the commonly used standard diffusion equation that models the action potential (AP) propagation, assumes the myocardium as a continuous domain (Keener and Sneyd, 1998), while in the real case, conduction in cardiac tissue is inherently discontinuous (Shaw and Rudy, 2010).

Fractional differential equations, that generalize the classical derivatives/integrals of to real or complex valued orders (Oldham and Spanier, 2006), gained incidence in several fields of applied mathematics (Ionescu et al., 2017; Machado and Kiryakova, 2017; Sun et al., 2018). Cardiac electrophysiological fractional models were recently reported able to characterize the ventricular repolarization expressed by a structurally heterogeneous myocardial domain (Bueno-Orovio et al., 2014). Although it is recognized that fractional derivatives/integrals can better describe experimental data, how to physically interpret the fractional order remains as an open problem whose answer is relative to the specific system under study. In Bueno-Orovio et al. (2014), the real valued fractional derivative is related with the average degree of tissular structural inhomogeneities. However, the estimation of the derivative order is bonded to the goodness of fit of the data. It would be desirable that a specific value of the derivative order could be translated to a tissular structure.

Fractal objects have been associated with distinct physical phenomena (Mandelbrot, 1982). The main feature of fractals is the self-similarity, meaning that the scaled parts resemble the whole, yielding to irregular patterns (Captur et al., 2016). Such patterns partially fill the embedding space, and in consequence a non integer dimension, or fractal dimension, describes the object. Thus, the overall morphologic complexity is measured by the fractal dimension (Bizzarri et al., 2011). Biological systems have been studied under the fractal perspective (Copley et al., 2012; Wedman et al., 2015; Lennon et al., 2016; Stankovic et al., 2016). There are reports suggesting that the fractal dimension discriminates between healthy and pathological conditions (Hiroshima et al., 2016; Zehani et al., 2016; Müller et al., 2017; Zhang et al., 2017; Popovic et al., 2018). In the cardiac context, structural remodeling generates significant fractal dimension variations (Zouein et al., 2014; Captur et al., 2016). Therefore, the fractal analysis could serve as a link between the geometrical complexity of the myocardium and the AP propagation dynamics under pathological conditions such as AF.

Bearing this ideas in mind, a new approach for assessing the effect of structural heterogeneous tissue on rotor dynamics based on complex fractional order derivatives is developed. We assume that the structurally remodeled myocardium undergoing CAF, has a fractal signature. Taking advantage of well developed mathematical theory, we relate the fractal dimension with the space fractional derivative order. It is evinced that complex valued orders arising if the fractal domain have the property of discrete-scale invariance. Previously, using a simplified cellular model, we found that the rotor stability is affected by real valued order derivatives (Ugarte et al., 2017). We extend the electrophysiological fractional system by means of complex order derivatives to assess the rotor dynamics and to implement a detailed atrial membrane formalism. The fractal structural heterogeneity of the tissue is then controlled by two parameters, namely the real and imaginary parts of the complex derivative. We test a set of discrete values for the complex derivative order and we analyze the stability of the rotors generated for each case. Our simulations include the standard diffusion solution given that it is a particular case of the new complex fractional order model.

## 2. Material and methods

### 2.1. Electrical potential over a fractal myocardium with discrete scale invariance

A fractal can be described by a fractional dimension and generalizes the Euclidean concept of integer space dimension (Mandelbrot, 1982). A fractal is a self-similar object and such property implies scale invariance. An object *f*(*x*) depending on the space variable *x*, is scale-invariant if after applying a scale factor ξ the following relation is obtained:

(1) $$\begin{array}{r} f\left(x\right)\sim \xi^{\gamma }f\left(\xi x\right), \end{array}$$

where γ is the fractal dimension. The scale factor ξ controls the scale of magnification applied. The value of γ indicates how the object fills the space and it is a measure of the object irregularity (heterogeneity). The fractal dimension γ can be real or complex: if the self-similarity is fulfilled only at discrete scales of observation (i.e., at discrete zoom factors), then γ is a complex number and the object is discrete-scale invariant. If self-similarity is preserved at the full range of scales, then γ is a real number, and the object is continuous-scale invariant. A mathematical description is given in the Supplementary Material, and for a detailed theoretical explanation please refer to Sornette (1998).

Let us assume the puntual current source *s*(*x*) over a discrete-scale invariant fractal myocardium. For simplicity, the myocardium is embedded on a one-dimensional space. We want to investigate, how the source *s*(*x*) interacts with the fractal myocardium. For this purpose, the convolution integral (2) over the bounded myocardium Ω is calculated, such as:

(2) $$\begin{array}{r} \phi \left(x\right)=\int_{\Omega }P\left(x-u\right)s\left(x\right)du, \end{array}$$

where *P*(*x*) is the normalized fractal structure-factor of the myocardium. If the Fourier image of *P*(*x*) converges for small and large values of the Fourier variable (Nigmatullin and Le Mehaute, 2005; Nigmatullin and Baleanu, 2013), then the function *P*(*x*) has the form:

(3) $$\begin{array}{r} P\left(x\right)=A_{0}{\text{Ψ}}_{\alpha }\frac{1}{|x{|}^{1-\alpha }}+A_{1}{\text{Ψ}}_{\alpha +j\beta }\frac{1}{|x{|}^{1-\alpha -j\beta }}+{\bar{A}}_{1}{\text{Ψ}}_{\alpha -j\beta }\frac{1}{|x{|}^{1-\alpha +j\beta }}, \end{array}$$

with:

(4) $$\begin{array}{r} {\text{Ψ}}_{\alpha \pm j\beta }=\frac{1}{\pi }\Gamma \left(1-\alpha \mp j\beta \right)\sin\left[\frac{\left(\alpha \pm j\beta \right)\pi }{2}\right], \end{array}$$

where Γ(·) is the Gamma function, *A*_0_ and *A*_1_ are complex constants, the operator $\bar{\cdot }$ represents the complex conjugation, α is the fractal dimension of domain *D*_Ω_ and β correspond to a log-periodic correction to the fractal dimension.

Each term in the right side of (3) corresponds to the Green function of the fractional Laplacian operator (Pozrikidis, 2016). Therefore, Equation (2) can be expressed as:

(5) $$\begin{array}{r} \phi \left(x\right)=\left[-A_{0}{\left(-\text{Δ}\right)}^{-\frac{\alpha }{2}}-A_{1}{\left(-\text{Δ}\right)}^{-\frac{\alpha +j\beta }{2}}-{\bar{A}}_{1}{\left(-\text{Δ}\right)}^{-\frac{\alpha -j\beta }{2}}\right]s\left(x\right), \end{array}$$

where ϕ(*x*) can be interpreted as the electrical potential over a fractal domain generated by a electrical source *s*(*x*). If the fractal myocardial structure is continuous-scale invariant, then the Laplacian complex-conjugates pair vanishes and the potential is governed by the real fractional Laplacian (recovering the model proposed in Bueno-Orovio et al., 2014). Equation (5) describes the electrical potential over a discrete-scale invariant fractal myocardium. In what follows, we will evaluate the effect of the Laplacian conjugates pair over the electrical potential in a structurally remodeled myocardium (i.e., a discrete-scale invariant fractal structure) under CAF conditions.

### 2.2. Complex fractional order diffusion model

We propose that the AP propagation in a structurally heterogeneous two-dimensional (2D) domain can be modeled by the following equation

(6) $$\begin{array}{r} \frac{\partial V}{\partial t}=\kappa \left(H_{x}^{\gamma }V+H_{y}^{\gamma }V\right)+\frac{1}{C}I, \end{array}$$

where *V* denotes the cellular membrane potential, *I* is the ionic transmembrane current, *C* is membrane capacitance, κ represents the diffusion coefficient assuming isotropic propagation, *x* and *y* are the spatial variables, *t* is the time variable, and γ = α + *jβ* is the complex fractional order. The operator $H_{x}^{\gamma }$ involves a pair of complex-conjugate derivative and is defined by:

(7) $$\begin{array}{r} H_{x}^{\gamma }=-\frac{1}{2}\left[{\left(-\frac{\partial^{2}}{\partial x^{2}}\right)}^{\gamma /2}+{\left(-\frac{\partial^{2}}{\partial x^{2}}\right)}^{\bar{\gamma }/2}\right], \end{array}$$

where $\bar{\gamma }=\alpha -j\beta$ is the complex conjugate of γ. The operator $H_{y}^{\gamma }$ in the Equation (6) is defined as in Equation (7) with respect to the variable *x*. The purpose on defining Equation (7) is to obtain a real-valued function after applying the complex order derivative (Machado, 2013; Hartley et al., 2016). Model (6) represents a generalization of the classical AP propagation model based on the standard diffusion operator (Trayanova et al., 2014).

### 2.3. Model of chronic atrial fibrillation

The Courtemanche atrial membrane formalism (Courtemanche et al., 1998) is used to calculate the term *I* in Equation (6). The ionic conductances are adjusted in order to implement the electrical remodeling due to CAF. According to experimental data (Van Wagoner et al., 1997; Bosch et al., 1999; Dobrev et al., 2001) we modify the maximum conductances of the transient potassium current (*I*_*to*_) and the L-type calcium current (*I*_*CaL*_) by a factor of 0.65, the maximum conductance of delayed rectifier potassium current (*I*_*Kur*_) is reduced by a factor 0.49, and the maximum conductance of the potassium time independent current (*I*_*K*1_) is incremented by a factor of 2.1.

Cholinergic activity is known as a factor that promotes CAF. The cholinergic effect is included in the Courtemanche model by implementing the acetylcholine-dependent potassium current (*I*_KACh_) (Kneller et al., 2002) and an acetylcholine concentration of 5 nM.

### 2.4. Stimulation protocol

Rotors are generated by applying the S1-S2 cross-field stimulation protocol. In this protocol, S1 is a train of stimuli with a basic cycle length of 400 ms and is applied to a boundary of the 2D domain, aiming to generate plane propagation waves. After S1, the S2 is a single premature stimulus applied at the lower quadrant that is adjacent to the S1 application boundary, when the last repolarization wave generated by S1 reaches the half of the domain. The coupling interval is measured as the difference between the time of the last S1 stimulus and the time when starting S2. A single stimulus is a rectangular wavefront current with a duration of 2 ms and an amplitude of twice the diastolic threshold. Restitution curves are calculated by applying a S1-S2 stimulation protocol. The S1 is a train of 10 stimuli with a basic cycle length (BCL) of 1000 ms, and S2 is the premature stimulus following S1. Measures of APD and CV are registered at the point located at two thirds of *L* from the stimulation point, provided that a wave of propagation is generated.

## 3. Atrial strand analysis

To assess the effect of β on the electrophysiological characteristics of the Courtemanche model, simulations over atrial strands are carried out. We use a set of test values for γ with α ∈ [1.1, 2] and β ∈ [0, 0.28], according to stability conditions (see Supplementary Material).

We perform the analysis at microscopic scale with measures from a cell within the strand, and at mesoscopic with representing properties of the atrial strand. At the microscopic scale, the transmembrane ionic currents and action potential duration (APD) from the middle cell are measured. At the mesoscopic scale, the APD dispersion (dAPD), spatial peak currents profiles and restitution curves are registered. The APD is defined at 90% of repolarization. The CV is measured between the points located at one third and two thirds of *L*. The global dAPD is defined as the range of the APD values within the strand. The local dAPD is negative and is defined as the difference between the minimum APD within the strand and the local APD. The peak ionic current spatial profiles are build using the peak values of the corresponding ionic current at each cell of the strand.

### 3.1. Reentry vulnerability and rotor dynamics analysis

The reentry vulnerability analysis is accomplished by applying the S1-S2 protocol at different coupling intervals. We define the vulnerable window (VW) as the difference between the maximum and minimum coupling intervals that triggers a rotor sustaining for at least two rotations within the domain.

The analysis of the rotor dynamics requires calculating the rotor tip trajectory from the phase maps (Bray et al., 2001). The phase analysis defines the rotor tip as the singularity point where the phase is undefined. A phase map is calculated, based on the values of *V* over the space domain at a given time. The Hilbert transform is obtained from each *V* time series, and the phase is calculated from the relation of the imaginary part of the Hilbert transform and the corresponding *V* value at a given time. The rotor dynamics is characterized by the motion of the singularity through the tissue.

### 3.2. Simulation setup

An atrial tissue is modeled as a 2D domain of 4 × 4 cm^2^ in surface and is discretized with uniform space steps of Δ*x* = Δ*y* = 321.5 μm. The time is discretized with a step of Δ*t* = 10^−2^ ms. The Equation (6) is numerically solved by splitting the operator (Marchuk, 1968; Strang, 1968). The complex order space derivative operator is calculated using a semi-spectral scheme (details are presented in the Supplementary Material). The time derivatives of the Courtemanche model are obtained using the explicit Euler approximation.

Initial conditions for the atrial tissue are set from unicellular simulations, where a single CAF remodeled cell is paced at a basic cycle length of 400 ms during 60 s. For assessing the rotors behavior for different degrees of structural heterogeneity, we define a set of test values for γ. The real part α is varied within the interval [1.1, 2] with a step of 0.1. The range of the imaginary part β is bounded to ensure numerical stability of Equation (6) (see Supplementary Material). Taking this into account, the imaginary part assumes two values β = {0, 0.28}. The diffusion coefficient κ is adjusted in order to generate plane propagation with a conduction velocity (CV) of 63 cm/s, when the CAF remodeling is not applied to the Courtemanche model. Therefore, a specific value of κ is defined for each α with β = 0.

Prior to the rotor simulations, the effect of β over the electrophysiological characteristics of the CAF model are studied using a 1D model. An atrial strand is modeled as a 1D domain with *L* = 2 cm discretized at *N* = 128 points. Simulations for a dynamical evolution during 10 seconds are executed. The stimulation is applied at the left side of the strand at a BCL of 1000 ms. Microscopic measures are registered from the middle cell of the strand.

## 4. Results

### 4.1. Atrial electrophysiological characteristics under complex order diffusion

#### 4.1.1. Microscale analysis

**Figure 1:**
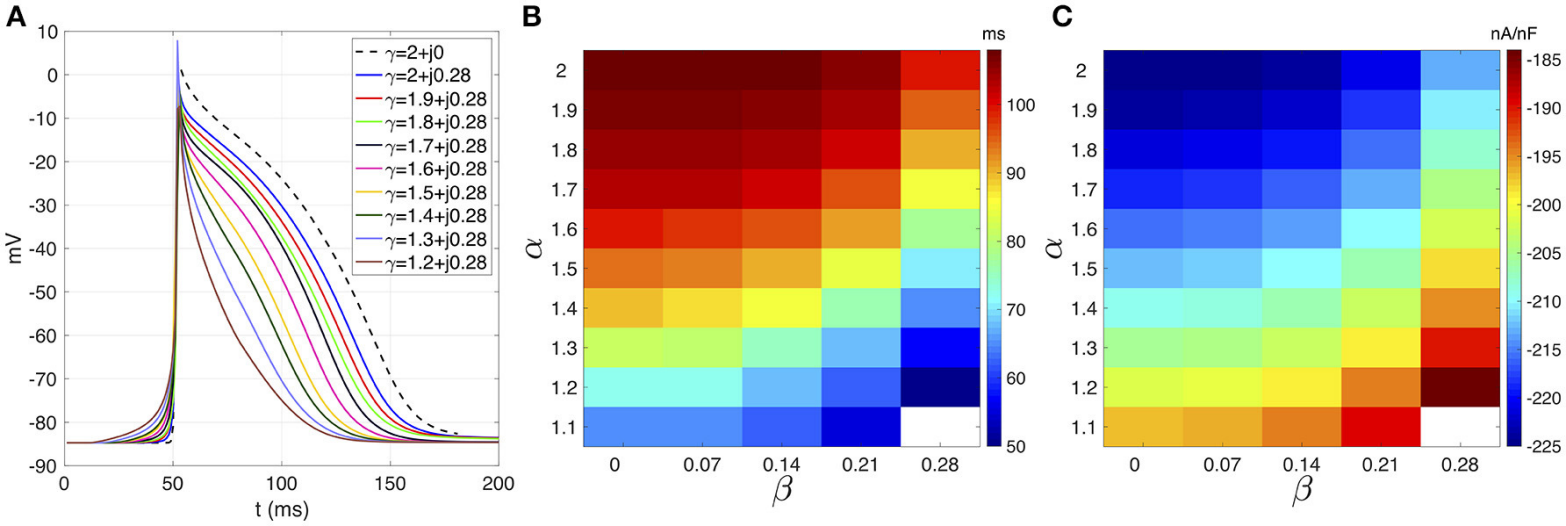
**(A)** Courtemanche's action potential registered at the center of the atrial strand under CAF conditions. Dashed line corresponds to standard diffusion and colored curves correspond to test values of γ. **(B)** APD map over the (α, β) plane. **(C)** Sodium peak current map over the (α,β) plane. White means that no record is obtained due to absence of propagation.

Figure 1A shows the action potentials (AP) registered from the middle cell within the strand for β = 0.28 fixed. The standard diffusion case (γ = 2 + *j*0, dotted line) is also shown. The AP foot during the depolarization phase describe a smoother transition from rest to activation as α decreases. The AP repolarization is also affected and reveals a decreasing APD as α decrease. In both cases, a significant difference between the standard diffusion and the new description with γ = 2 + *j*0.28 is observed. This is clarified in Figure 1B, that presents the APD map over the complex plane (α, β). The APD decrease smoothly as α decreases and β increases. However, β generates greater reductions of the APD than α. For example, a reduction of 9 ms is achieved by fixing α = 2 and increasing β from 0 (APD = 108 ms) to 0.28 (APD = 99 ms). In order to generated such an APD reduction with β = 0, a reduction from 2 to 1.6 is needed.

**Figure 2:**
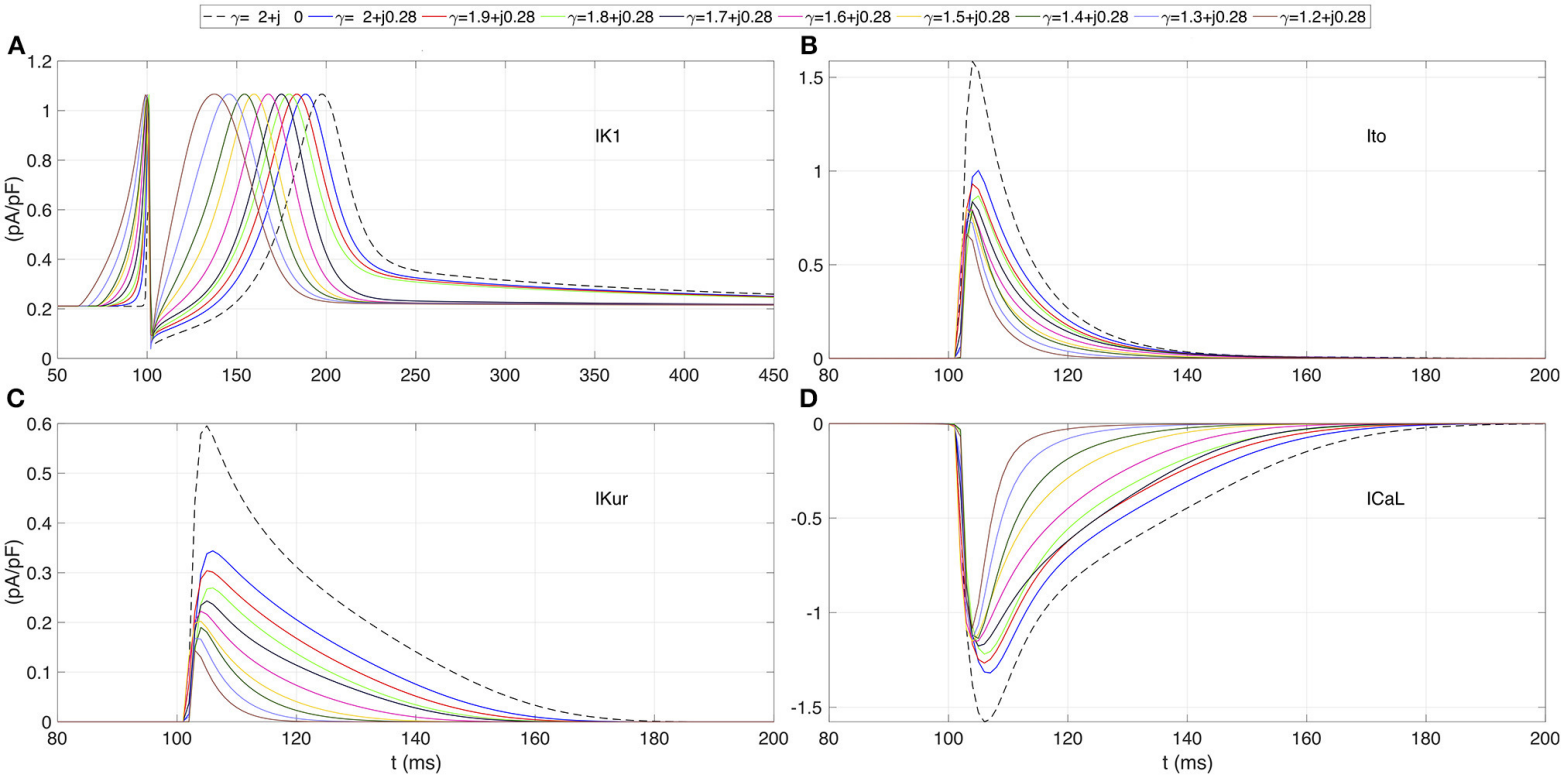
Transmembrane ionic currents registered at the center of the atrial strand for several test values of γ. **(A)** Rectifier potassium current *IK*1. **(B)** Outward transient potassium current *Ito*. **(C)** Ultrarapid delayed rectifier potassium current *IKur*. **(D)** L-type calcium current *ICaL*. Dashed line corresponds to standard diffusion and colored curves correspond to test values of γ.

The effects of β on the ionic currents are shown in Figures 1C, 2. Figure 1C depicts the map of the sodium peak current. Reducing α, or increasing β, increases the peak value, but the effect is more significant for β increments. Figure 2 illustrates the effect of γ over the temporal series of representative ionic currents. Complex values of γ modulate the ionic current transients which is in accordance with the modulations observed during the despolarization and repolarization phases of the atrial AP.

#### 4.1.2. Mesoscale analysis

**Figure 3:**
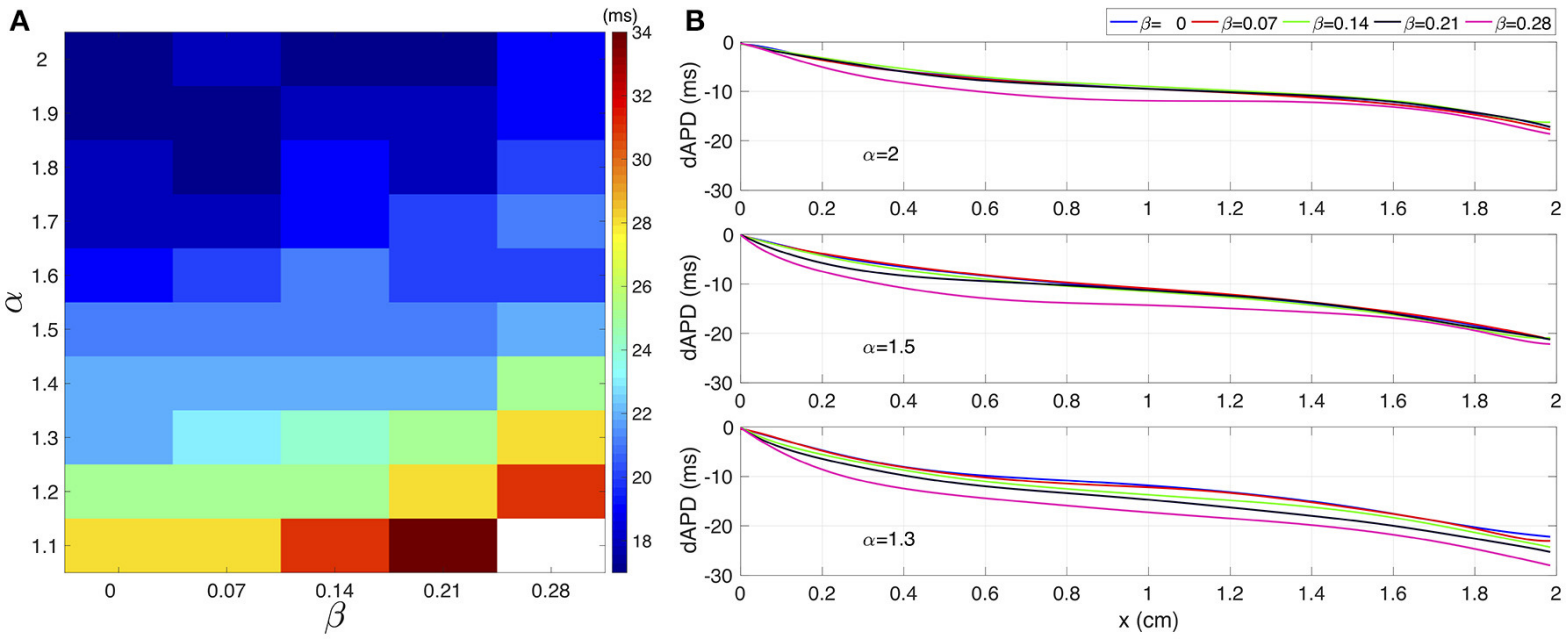
**(A)** Global dAPD map over the (α,β) plane. **(B)** Local dAPD spatial profiles for three distinct values of α. In each panel, for a given α, the β value varies between 0 and 0.28.

Figure 3 shows the effect of γ over the dAPD. Figure 3A shows the map of the global dAPD over the complex plane (α, β). The map suggests that the global dAPD increase with β and with decrements of α. Although the β modulation is stronger, the dAPD changes are smaller in magnitude than those observed in the microscopic analysis. Figure 3B shows the local dAPD spatial profiles for three representative values of α. For α fixed, β modulates the local dAPD profiles, producing relevant changes for small α values.

**Figure 4:**
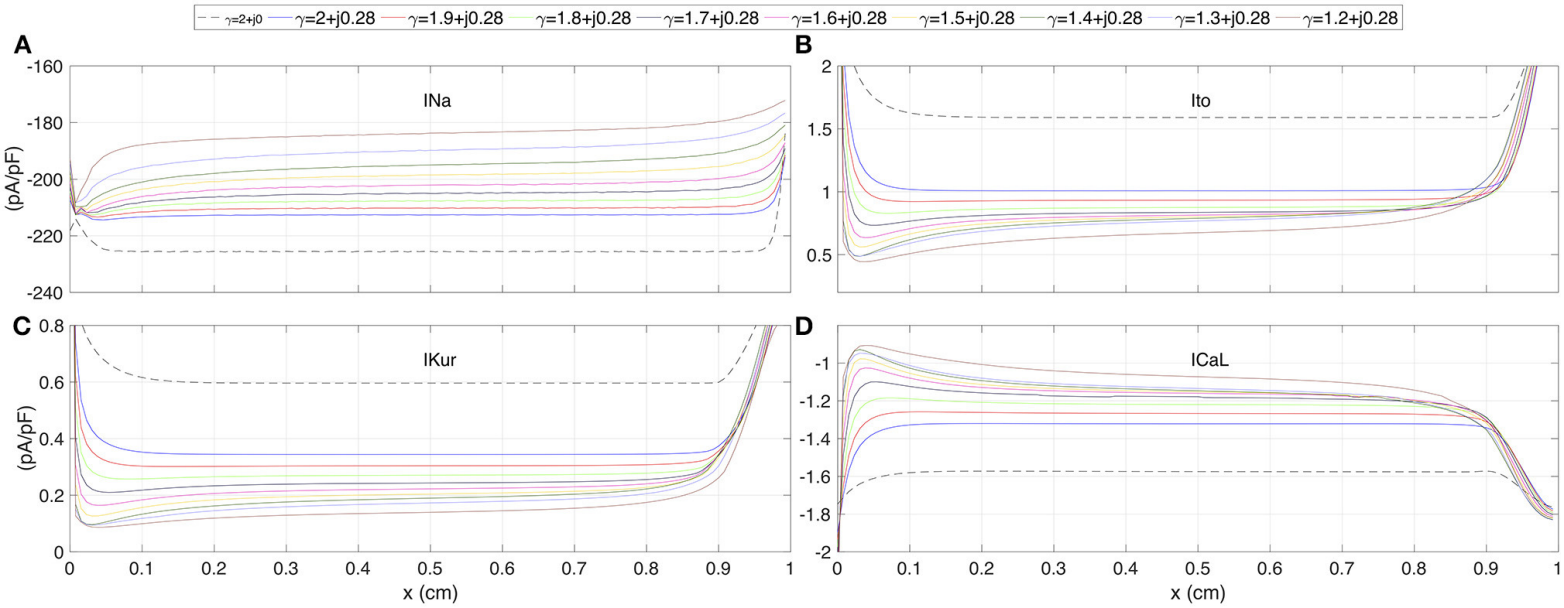
The spatial profiles of ionic current peaks for several test values of γ. **(A)** Sodium current *INa*. **(B)** Outward transient potassium current *Ito*. **(C)** Ultrarapid delayed rectifier potassium current *IKur*. **(D)** L-type calcium current *ICaL*. Dashed line corresponds to standard diffusion and colored curves correspond to test values of γ.

The spatial profiles of ionic current peaks for *INa*, *Ito*, *IKur*, and *ICal* are illustrated in Figure 4. Complex values of γ generate a family of spatial profiles with differences in magnitude. Note the gap in magnitude between the profiles family generated with complex values of γ and the profile for the standard diffusion case (γ = 2 + *j*0, dashed line).

**Figure 5:**
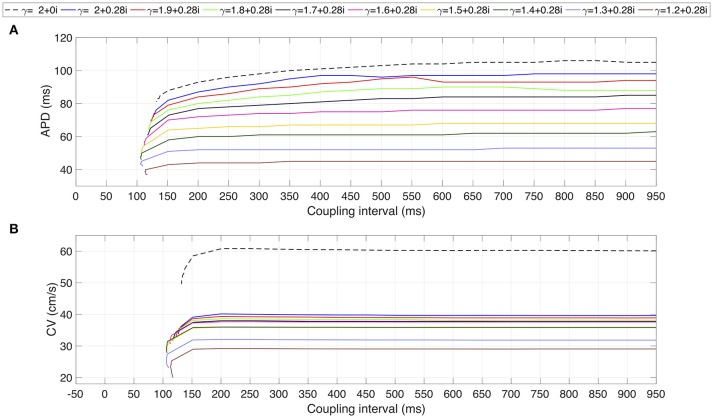
Restitution curves for several test values of γ. **(A)** APD restitution curve. **(B)** CV restitution curve. Dashed line corresponds to standard diffusion and colored curves correspond to test values of γ.

Finally, the restitution curves are shown in Figure 5. As it is characteristic of CAF, flat curves for APD and CV are generated for complex values of γ. The coupling interval values for each family of restitutions curves suggest a non linear behavior: (i) in the interval 2 ≥ α > 1.2, for decreasing values of α, the atrial strand generates propagation with premature stimulus; (ii) for α < 1.3 premature stimulation is accepted for increasing values of α. For the CV restitution curves, there is a notorious difference in magnitude between the standard diffusion case and the family of curves generated with γ complex. This difference is not evident for the APD restitution curves.

### 4.2. Rotor simulations

**Figure 6:**
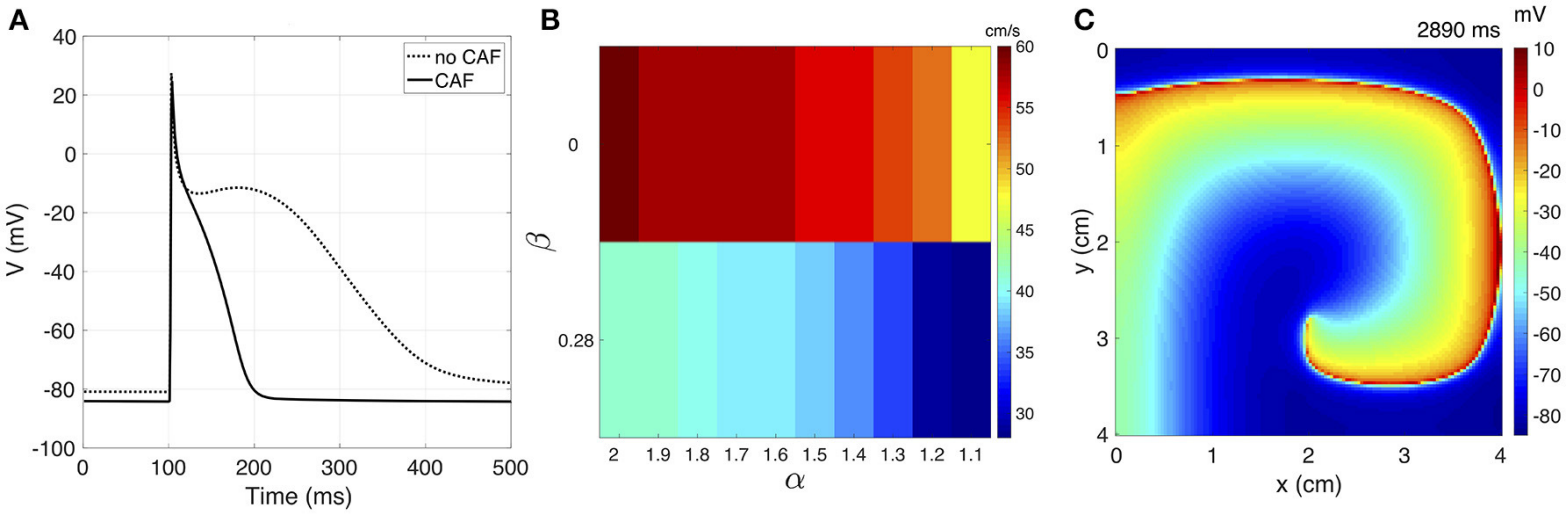
**(A)** The unicellular AP curves for no CAF and CAF models. **(B)** CV map over the complex plane (α, β) under CAF conditions. **(C)** Sample frame of rotor propagation for γ = 1.6 + *j*0.28.

We present the results of rotor simulations using the novel model based on the complex fractional order diffusion operator. In a unicellular environment, pacing is applied during 60 s to the Courtemanche model before and after applying the CAF electrical remodeling. The last AP generated for each case are shown in Figure 6. Modifications in the AP characteristics resulting from the CAF conditions can be summarized as: the action potential duration reduces from 309 ms to 96 ms and the resting membrane potential decreases from −80.98 mV to −84.67 mV.

We calculate κ for each variation of γ = α + *j*0 for generating a CV of 63 cm/s without CAF. Table 1 shows the adjusted κ values and the corresponding CV for both physiological conditions. The CAF remodeling reduces the CV as α decreases or β increases. This behavior is depicted in the bar graph of Figure 6. The propagation generated with standard diffusion decreases the CV by 5%, while for γ = 2 + *j*0 and γ = 1.2 + *j*0.28 the CV decreases by 25% and 55%, respectively. For γ = 1.1 + *j*0.28 propagation cannot be generated under CAF conditions.

**Table 1:** Adjusted values of κ that yield propagation at 63 cm/s under non-CAF conditions and β = 0. CV (cm/s) for CAF conditions with β = {0, 0.28}.

	**α**	**2**	**1.9**	**1.8**	**1.7**	**1.6**	**1.5**	**1.4**	**1.3**	**1.2**	**1.1**
	**κ(cm^2^/s)**	**0.40**	**0.59**	**0.87**	**1.26**	**1.84**	**2.68**	**3.81**	**5.38**	**7.52**	**10.28**
CAF	CV, β = 0	60.10	57.87	57.87	57.87	57.87	55.80	55.80	53.88	52.08	47.35
	CV, β = 0.28	41.11	41.11	40.06	39.06	39.06	38.11	36.34	33.97	28.94	–

*The mark–means no sustained propagation*.

#### 4.2.1. Vulnerable window

The analysis of the reentry vulnerability of the tissue is performed by applying the S1–S2 protocol for each variation of γ and measuring the VW as detailed above. Under this scheme, we were not able to generate a sustained reentry for any couple interval value for γ = {1.2 + *j*0.28, 1.1 + *j*0.28}. Table 2 shows the results. For β = 0 the VW increases as α increases. For β = 0.28 the VW tends to increase as α decreases, reaching a maximum with α = 1.4. The values of VW obtained with β = 0.28 are larger than those obtained with β = 0.

**Table 2:** Values of VW (ms) measured for several test values of γ after applying the S1–S2 cross-filed stimulation protocol to the tissue.

	α									
**β**	**2**	**1.9**	**1.8**	**1.7**	**1.6**	**1.5**	**1.4**	**1.3**	**1.2**	**1.1**
0	16	16	16	17	18	21	18	24	31	36
0.28	39	38	38	39	40	42	49	0	0	0

#### 4.2.2. Rotor dynamics

**Figure 7:**
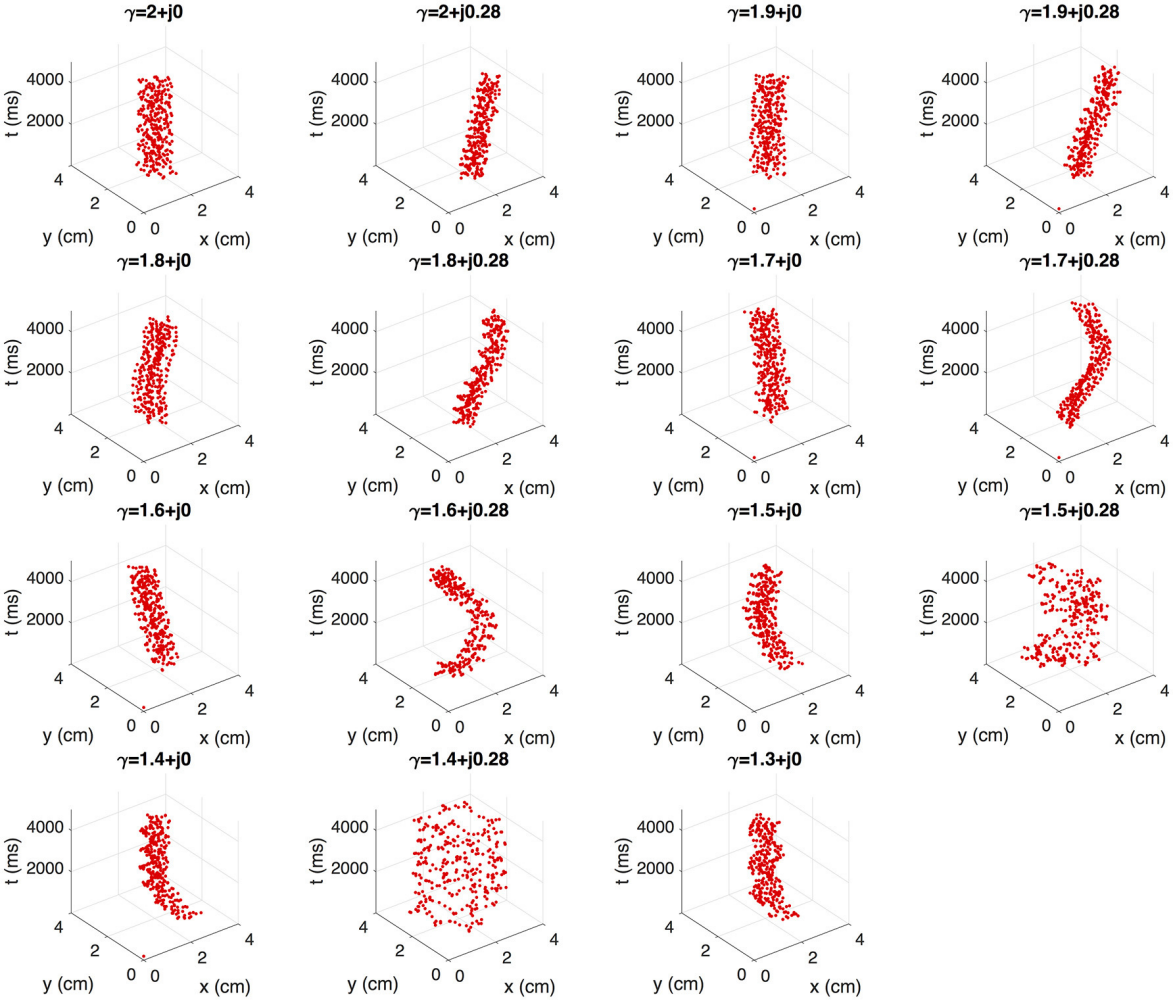
Effect of γ on the rotor dynamics represented by singularity cores.

**Figure 8:**
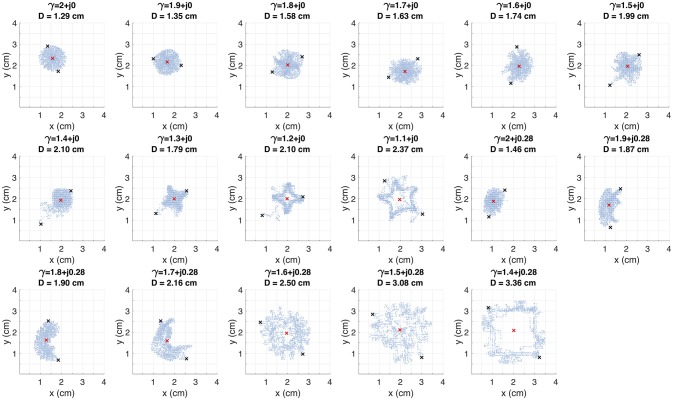
Trajectory of the rotor tip within the spatial plane (*x, y*) for several test values of γ. The red mark indicates the rotor core. The *D* value corresponds to the distance between the black marks. These marks depict the farthest points within the trajectory.

Fibrillation episodes in the 2D atrial tissue are simulated by generating rotor mechanisms. We apply the S1–S2 protocol for simulating fibrillatory episodes of 5 seconds. The variations of γ = α + *j*0.28 with α ≤ 1.3 are excluded since propagation cannot be sustained. We study the effect of γ on the rotor tip trajectories through the phase singularity motion. Figure 7 shows the filament of the phase singularity, that represents the temporal evolution of the rotor tip depicted in the tridimensional space (*x, y, t*). The filament corresponding to the standard diffusion case (γ = 2 + *j*0) stably evolves around an axis parallel to the *t*-axis. By varying α and β, the filaments describe distinct spatially complex trajectories. In order to quantify the effect of γ over the rotor spatial stability, we estimate the tip maximal displacement (*D*) as the maximum euclidean distance between two points that belongs to the singularity filament, assuming all points as coplanar:

(8) $$\begin{array}{r} D=\max\left\{\sqrt{{\left(x_{j}-x_{k}\right)}^{2}+{\left(y_{j}-y_{k}\right)}^{2}}\right\}, \end{array}$$

where (*x*_*j*_, *y*_*j*_) and (*x*_*k*_, *y*_*k*_) are any couple of points within the filament. Figure 8 shows the filaments within the same plane (*x, y*). The red mark represents the core of the rotor. The black marks represent the farthest points within the filament, where the distance between them is equal to *D*. The standard diffusion generates the lowest value of *D* that can be interpreted as the most stable rotor dynamics among the γ variations. For β = 0, the value of *D* tends to increase as α decreases, depicting filaments with closed trajectories around the core of the rotor. For β = 0.28, different dynamics can be identified: for α > 1.6 the rotor meanders without defining a stable core, and for α ≤ 1.6 the rotor meandering describe closed paths around an stable core. Note that the values of *D* with β = 0.28 are greater than their counterparts with β = 0.

## 5. Discussion

The present computational study assesses the interplay between rotors and the structural heterogeneity of a CAF remodeled tissue. The structural remodeled tissue is modeled as fractal structure with discrete-scale invariance. Such structural heterogeneities are implemented using the complex derivative order γ = α + *jβ*, that represents the fractal dimension of the domain with log-periodic corrections. Thus, the proposed complex fractional order diffusion equation has two degrees of freedom (α and β) besides those imposed by the cellular model (reactive term) and the diffusion coefficient (κ). Having the same electrical remodeling due to CAF, through the simulations we found that: the incremental changes of β generates electrophysiological modulation to a greater extent than the decremental changes of α. The quantitative and qualitative changes in the repolatization features (such, as APD, and ionic currents) generated by β are more visible on the microscale. At the mesoscale, the extent of β modulation depends on α. The observed restitution properties indicate that complex values of γ favors the premature propagation. This result suggests that increasing structural complexity of a discrete-scale invariant atrial strand, has proarrhythmic effects which is characteristic of CAF. The reentry vulnerability of the tissue can be modulated by γ, where the shortest VW achieved corresponds to the standard diffusion case. Furthermore, the extent in which the CAF remodeling reduces the CV depends on the value of γ, having the smaller reduction with the standard diffusion. Lastly, by varying γ the rotor dynamics is affected, generating meandering or drifting rotors, and modifying the area covered by the rotor core.

### 5.1. The complex order model of AP propagation

Fractal analysis and fractional differential equations have proven to be useful tools for describing real processes (Mandelbrot, 1982; Captur et al., 2016; Ionescu et al., 2017; Machado and Kiryakova, 2017). Although, there is a general agreement about a relation between both theories, the formal mathematical arguments supporting this relation are still being developed. Important advances in this regard have been made in the last two decades (Sornette, 1998; Nigmatullin and Le Mehaute, 2005; Nigmatullin and Baleanu, 2013; Calcagni, 2017; Nigmatullin et al., 2017). Therefore, this work contextualizes this theoretical frame and situates it within the scope of cardiac electrophysiological systems. A complex fractional order diffusion equation is proposed considering the propagation medium as a fractal object. The complex derivative order implies that the myocardium is discrete-scale invariant. Such a property is characteristic of, for example, fractal trees, percolation and diffusion-limited aggregates (Sornette, 1998). These mathematical objects have been applied to describe cardiovascular components: (i) fractal trees are used to study the human coronary vasculature (Zamir, 1999; Zenin et al., 2007), the His-Purkinke conduction system (Goldberger and West, 1987; Berenfeld, 1991), and the atrial pectinate musculature (Goldberger and West, 1987; Goldberger et al., 1990); (ii) percolation clusters serve for modeling fibrosis (Vigmond et al., 2016), and a heterogeneous and discrete myocardium (Alonso and Bär, 2013); (iii) diffusion-limited aggregates are used to model fibroblast (Dickinson et al., 1994; Nogueira et al., 2011). The atrial tissue is composed by a discrete net of cardiomyocytes, a microscopic structure of capillaries, and non-myocyte cells such as the fibroblasts. This complex atrial architecture can be considered as a fractal object, whose mechanisms lead to discrete-scale invariance. Moreover, the fractal analysis has been applied to characterize structural pathological states (Cross, 1997; Fuseler et al., 2007; Zouein et al., 2014; Captur et al., 2015, 2016). Thus, the complex order diffusion equation can serve as a good model of atrial structural remodeling.

Previous report of fractional electrophysiological model of cardiac propagation (Bueno-Orovio et al., 2014), justified the adoption of real fractional derivative order as a degree of structural heterogeneity between a homogeneous domain, dictated by the standard diffusion model (α = 2, β = 0), and the domain inhomogeneities, dictated by the ballistic regime (α = 1, β = 0). We want to stress here that our approach does not disagree with the one proposed in Bueno-Orovio et al. (2014). Indeed, a fractal dimension of γ = 2 +*j*0 corresponds to a full homogeneous domain. As the fractal dimension decreases (α → 1), the irregularity of the domain increases (i.e., the increasing structural heterogeneities). Thus, the transition between both regimes is preserved. A purely real derivative order would imply a system with no characteristic scale, and a given property is held regardless the scale of observations, also referred as a scale-free system. However, in real systems, physical cut-offs prevent the invariance spreading over all scales Khaluf et al. (2017). Therefore, the complex order derivative yields a more realistic model of electrophysiological systems. The inclusion of the imaginary part β implies the existence of relevant length scales within the electrophysiological system (Sornette, 1998), one of these scales may be related with the size of a single cardiomyocyte. Through the complex derivative order, the characteristic scales that may play a role during the cardiac dynamics can be identified. For this reason, our complex order model extends the real fractional model, in order to enhance the comprehension of the cardiac structure effects on cardiac propagation.

### 5.2. The modulation of electrophysiological properties through the complex fractional derivative

We evinced that the depolarization phase of the AP is modulated by complex derivative order. The slow down of the AP foot was related to structural heterogeneities in atrial and ventricular tissue from adult dogs (Spach et al., 1998). This observation agrees with the results obtained using the complex order diffusion model.

It is well recognized that the APD is reduced under CAF conditions due to the electrical remodeling (Wijffels et al., 1995; Bosch et al., 1999; Workman et al., 2001). Our results suggest that structural changes reduce the APD under CAF conditions. Experimental measures of APD reduction were obtained mainly from unicellular patch clamp experiments. Determining the APD response to structural remodeling is a difficult task. In fact, there are some experimental studies reporting, through subrogate measures, that the APD decreases in patients undergoing CAF and structural remodeling (Morillo et al., 1995; Graux et al., 1998; Vasquez et al., 2010) and, therefore, our results agree with those observations.

Clinical studies described different behaviors for the dAPD in CAF patients, indicating a global increment of the dAPD (Boutjdir et al., 1986) and also reporting a regional dependance (Kamalvand et al., 1999). Our results suggest that, under similar electrical remodeling conditions, distinct degrees of global dAPD can be generated by means of γ. This can be used to represent different regions within the atria, which coincides with the description of Kamalvand et al. (1999). Furthermore, the local dAPD profiles obtained by means of the complex order model indicates a reduction of the APD in the direction of propagation. This gradient has been observed in rabbit atria, sinoatrial node zone (Boyett et al., 1999), rat ventricular cellular cultures (Badie and Bursac, 2009) and human ventricles (Hanson et al., 2009).

The reduction of CV plays an important role in the onset of AF, because it can favor the occurrence of reentry (Bosch et al., 1999). The structural remodeling under CAF conditions is a factor that alters the CV (Jalife and Kaur, 2014; Nattel and Harada, 2014). Our results agree with those observations: decreasing CV values are obtained when α decreases or β increases and their combination represents different degrees of structural heterogeneities. The changes in CV result from varying the complex order γ under the same CAF electrical remodeling conditions, where the reductions in CV are greater when increasing the imaginary part β. Local structural characteristics in the atria are important to determine the propagation of the AP, meaning that the CV varies according on the region (Lesh et al., 1996). When the CAF electrical remodeling occurs, the extent of CV deterioration depends on the atrial zone (Markides et al., 2003; Xia et al., 2004; Lalani et al., 2012). Using the complex fractional model it is possible to simulate different atrial tissues with heterogeneous CV values that can represent distinct atrial regions.

### 5.3. Rotor dynamics

The focal ectopic activity is an important source of reentrant propagation (Haïssaguerre et al., 1998; Arora et al., 2003). There is experimental evidence suggesting that the mechanisms determining the tissue vulnerability to reentry are related with structural and electrical remodeling interactions (Narayan et al., 2017). The complex fractional model reveals that increasing the degrees of structural heterogeneity increase the VW. Under the conditions of our simulations, these results indicate that increased vulnerability to reentry might be regulated by the underlying tissular structure. Additionally, we show that the VW values increase significantly with the imaginary part, so that β can be interpreted as a parameter representing a specific type of structural complexity.

The interest in rotor dynamics research has increased since clinical reports claim that rotor ablation improves the rates of success in human CAF treatment (Narayan et al., 2012). Using in silico models, mechanistic explanations and therapeutic approaches have been tested (Zhao et al., 2015; Berenfeld, 2016; Guillem et al., 2016; Tobón et al., 2017). In this work, we assessed the effect of structural heterogeneities on rotor propagation by varying the complex derivative order γ. For real values of γ (i.e., γ = α + *j*0) meandering quasi-stable rotors, with a shape-changing core, are generated. For complex values of γ (i.e., γ = α + *j*0.28) the rotor dynamics varies from drifting trajectories (α > 1.6) to meandering trajectories whose core is markedly greater with respect to those with β = 0. As mentioned above, the inclusion of the imaginary part β yields a major modulation of electrophysiological features. In the case of the standard diffusion (i.e., γ = 2 + *j*0), corresponding to a structurally homogeneous tissue (Bueno-Orovio et al., 2014), the rotor meanders in a quasi-stable form and describes the minimum tip displacement among all γ variations. Therefore, by varying γ, the spatial stability of the rotor can be modulated, obtaining a wide range of rotor dynamics. Previous simulation studies report quasi-stable dynamics using the Courtemanche model (Cherry and Evans, 2008; Wilhelms et al., 2013). Our standard diffusion simulations agree with those reports. However, additionally we evinced that using the CAF remoled Courtemanche formalism and keeping its parameters fixed, unstable rotor trajectories can ben obtained through γ variations. Thus, we show that using the complex fractional order diffusion model, it is possible to simulate realistic CAF conditions in which meandering and drifting rotors are observed. These dynamics can be related with increasing degrees of structural heterogeneity causing difficulties in accurately locating and ablating rotors. Under such conditions the CAF may be not reverted which agrees with experimental observations (Buch et al., 2016; Steinberg et al., 2017).

The growing evidence that structural remodeling has a relevant role in AF dynamics, leads the computational electrophysiology researchers to incorporate myocardial structure features in the AP propagation models. There is a lack of consensus on defining how purely structural properties has to be included in a computational model. Variable diffusion tensor (ranging from reduced conductivity to zero conductivity), boundary conditions and non-cardiomyocite active models, are proposals for modeling the myocardial structure (Trayanova et al., 2014; Brown et al., 2015). There is a common element among these approaches: the standard diffusion equation that assumes the cardiac tissue as a continuum (Keener and Sneyd, 1998). Although these computational schemes have improved our knowledge about the CAF mechanisms, modeling the inherently discontinuous myocardium as a continuous domain is inconsistent with the cardiac histological structure (Shaw and Rudy, 2010). In this work, the cardiac structural heterogeneity is implemented using a complex conjugate derivative operator. The resulting mathematical model generalizes the previously established propagation models (Keener and Sneyd, 1998; Bueno-Orovio et al., 2014) without the restriction of assuming a homogeneous domain.

### 5.4. Limitations

The biophysical interpretation of the complex derivative order is build on the mathematical theory of smoothed functions averaged over fractal sets (Nigmatullin and Le Mehaute, 2005). This problem has been solved for fractional temporal derivatives and the relation with the complex dimension has been stablished (Nigmatullin et al., 2017). This theory is supported by experimental results (Nigmatullin et al., 2007, 2018). However, the solution for spatial derivative operators is a more complex problem and the averaging procedure only admits specific types of fractals (Nigmatullin and Baleanu, 2013). Therefore, although in this work a fractal as medium of propagation was assumed, the specific fractal that is related with the fractional Laplacian operator has not been stablished. Future investigations will look into this problem. The simulations are performed in 2D domains. Nevertheless the atrial tissue is a 3D complex domain, whose heterogeneities can impact the rotor dynamics (Kharche et al., 2015). For this reason, our results cannot be generalized until analyzing 3D experiments of rotor propagation. The lack of detailed experimental data describing the spatial distribution of atrial electrophysiological properties does not allow an exhaustive assessment of the simulation outcomes. Therefore, the results obtained in this work invite to design experimental setups in order validate them.

## 6. Conclusions

The new complex fractional order diffusion model allows the simulation of a wide range of rotor dynamics. The results can be correlated with changes in the atrial tissular structure that have been observed in the clinical practice. This approach is a step toward an integral electrophysiological mathematical model that embodies the structural and electrical features of the myocardium.

## Author contributions

All authors contributed to conception and design of the study; JU performed the simulations. JU and CT wrote the first draft of the manuscript. All authors contributed to manuscript revision, read and approved the submitted version.

### Conflict of interest statement

The authors declare that the research was conducted in the absence of any commercial or financial relationships that could be construed as a potential conflict of interest.
