# Supplementary material for: Atrial Rotor Dynamics Under Complex Fractional Order Diffusion
Source: Front Physiol. 2018 Jul 24;9:975. doi: 10.3389/fphys.2018.00975 (PMC6066719; doi:10.3389/fphys.2018.00975)
Supplement: Supplementary file 1 [file Presentation_1.PDF]

frontiers

Supplementary Material: Atrial rotor dynamics under complex fractional order diffusion

1 NUMERICAL RESOLUTION OF THE COMPLEX CONJUGATES PAIR FRACTIONAL DIFFUSION OPERATOR

The complex fractional order diffusion model over a spatial domain $\Omega : (x, y) \in \mathbb{R}^2 : [0, L_x] \times [0, L_y]$ with Neumann boundary conditions is:

$$\frac{\partial V}{\partial t} = \kappa \left(H_x^{\gamma} V + H_y^{\gamma} V \right) + \frac{1}{C} I, \tag{S1}$$

The equation (S1) is solved by applying the splitting operator scheme (Marchuk, 1968; Strang, 1968). The reactive I and diffusive terms are integrated separately. The term involving the complex order operator is expressed as follows:

$$\frac{\partial V}{\partial t} = -\frac{\kappa}{2} \left[\left(-\frac{\partial^2}{\partial x^2} \right)^{\gamma/2} + \left(-\frac{\partial^2}{\partial x^2} \right)^{\overline{\gamma}/2} \right] V - \frac{\kappa}{2} \left[\left(-\frac{\partial^2}{\partial y^2} \right)^{\gamma/2} + \left(-\frac{\partial^2}{\partial y^2} \right)^{\overline{\gamma}/2} \right] V. \tag{S2}$$

The semi-spectral approach for numerical solution of the space-fractional Laplacian previously proposed (Bueno-Orovio et al., 2014), is adapted here. Discretizing the spatial domain Ω in N^2 spatial nodes, Eq. (S2) yields:

$$\begin{aligned} \frac{\partial \tilde{V}(k_x, k_y, t)}{\partial t} &= -\frac{\kappa}{2} \left(\lambda_{k_x}^{\gamma} + \lambda_{k_x}^{\overline{\gamma}} \right) \tilde{V}(k_x, k_y, t) - \frac{\kappa}{2} \left(\lambda_{k_y}^{\gamma} + \lambda_{k_y}^{\overline{\gamma}} \right) \tilde{V}(k_x, k_y, t), \\ &= -\kappa \lambda_{k_x}^{\alpha} \cos(\beta \log \lambda_{k_x}) \tilde{V}(k_x, k_y, t) - \kappa \lambda_{k_y}^{\alpha} \cos(\beta \log \lambda_{k_y}) \tilde{V}(k_x, k_y, t), \end{aligned} \tag{S3}$$

where $\lambda_{k_x} = k\pi / L_x$, $k = 0, 1, \ldots, N-1$, are the eigenvalues of the Laplacian operator, $\tilde{V}(k_x, k_y, t)$ is the discrete 2D cosine spatial transform of V and L_x, L_y are the lengths of the domain in the x and y direction. The time derivative is solved in the frequency domain using the Euler implicit scheme:

$$\begin{aligned} \frac{\tilde{V}(k, t+\Delta t) - \tilde{V}(k, t)}{\Delta t} &= -\left[\kappa \lambda_{k_x}^{\alpha} \cos(\beta \log \lambda_{k_x}) + \kappa \lambda_{k_y}^{\alpha} \cos(\beta \log \lambda_{k_y}) \right] \tilde{V}(k_x, k_y, t+\Delta t), \\ \tilde{V}(k, t+\Delta t) &= \frac{1}{1 + \kappa(\Delta t) \left[\lambda_{k_x}^{\alpha} \cos(\beta \log \lambda_{k_x}) + \lambda_{k_y}^{\alpha} \cos(\beta \log \lambda_{k_y}) \right]} \tilde{V}(k, t). \end{aligned} \tag{S4}$$

The numerical stability of Eq. (S4) is conditioned by:

$$\left| 1 + \kappa(\Delta t) \left[\lambda_{k_x}^{\alpha} \cos(\beta \log \lambda_{k_x}) + \lambda_{k_y}^{\alpha} \cos(\beta \log \lambda_{k_y}) \right] \right| \geq 1. \tag{S5}$$

If $L_x = L_y = L$, then $\lambda_{k_x} = \lambda_{k_y} = \lambda_k$. Thus, the cosine functions in S5 can be added. We consider the positive values of the cosine function, for $\alpha > 0$:

$$\begin{aligned} \cos(\beta \log \lambda_k) &\geq 0 \\ \beta \log \lambda_k &\leq \frac{\pi}{2} \\ 0 \leq \beta &\leq \frac{\pi}{2\log(\lambda_k)}, \end{aligned} \tag{S6}$$

that represents the stability condition of solution (S4). Each eigenvalue λ_k must satisfy inequality (S6). For N discretized nodes, the interval of variation of β can be defined using the largest eigenvalue λ_{N-1}.

2 DISCRETE-SCALE INVARIANT FRACTALS

A fractal can be described by a fractional dimension and generalizes the Euclidean concept of integer space dimension (Mandelbrot, 1982). A fractal is a self-similar object and such property implies scale invariance. Mathematically, a function $f(x)$ depending on space variable x, is scale-invariant if there is a number $\mu(\xi)$ fulfilling:

$$f(x) = \mu f(\xi x), \tag{S7}$$

where ξ is the scale factor. The solution of equation (S7) is the power law $f(x) \sim x^{\gamma}$, where $\gamma = -\log\mu/\log\xi$ is the fractal dimension.

If a set of continuous values of ξ obeys equation (S7), then the system $f(x)$ is continuous-scale invariant and $\gamma \in \mathbb{R}$. On the contrary, if equation (S7) is met only for a discrete set of values $\xi_n = \xi^n$ ($n = 0, \pm1, \pm2, \ldots$), then the system $f(x)$ is discrete-scale invariant. Under this discrete-scale condition, and applying the power law into equation (S7) (Sornette, 1998):

$$\gamma = -\frac{\log\mu}{\log\xi} + j\frac{2\pi n}{\log(\xi)}, \tag{S8}$$

with $j = \sqrt{-1}$. Equation (S8) implies that the fractal dimension is a complex number. If $n = 0$, then the continuous-scale invariance is recovered. Therefore, the complex dimension is a generalization corresponding to discrete-scale invariance (Sornette, 1998). Complex dimensions involve a log-periodic modulation of the leading power law $x^{\Re e\{\gamma\}}$, that is fundamental in establishing a relation between the fractal dimension and the order of fractional derivative/integral operators (Nigmatullin and Le Mehaute, 2005; Nigmatullin and Baleanu, 2013; Sornette, 1998).

REFERENCES

Bueno-Orovio, A., Kay, D., Grau, V., Rodriguez, B., Burrage, K., and Interface, J. R. S. (2014). Fractional diffusion models of cardiac electrical propagation : role of structural heterogeneity in dispersion of repolarization. *Journal of the Royal Society Interface* 11

Mandelbrot, B. (1982). *The Fractal Geometry of Nature*. Einaudi paperbacks (1997)

Marchuk, G. I. (1968). On the construction and comparison of difference schemes. *Aplikace matematiky* 13, 103–132

Nigmatullin, R. R. and Baleanu, D. (2013). New relationships connecting a class of fractal objects and fractional integrals in space. *Fractional Calculus and Applied Analysis* 16, 911–936. doi:10.2478/s13540-013-0056-1

Nigmatullin, R. R. and Le Mehaute, A. (2005). Is there geometrical/physical meaning of the fractional integral with complex exponent? *Journal of Non-Crystalline Solids* 351, 2888–2899. doi:10.1016/j.jnoncrysol.2005.05.035

Sornette, D. (1998). Discrete-scale invariance and complex dimensions. *Physics Report* 297, 239–270. doi:10.1016/S0370-1573(97)00076-8

Strang, G. (1968). On the construction and comparison of difference schemes. *Journal of Numerical Analysis* 5, 506–517
